# Evaluation of a new arterial pressure-based cardiac output device requiring no external calibration

**DOI:** 10.1186/1471-2253-7-9

**Published:** 2007-11-09

**Authors:** Christopher Prasser, Sylvia Bele, Cornelius Keyl, Stefan Schweiger, Benedikt Trabold, Matthias Amann, Julia Welnhofer, Christoph Wiesenack

**Affiliations:** 1 Department of Anesthesiology, University Hospital Regensburg, Franz-Josef-Strauß-Allee 11, Regensburg, 93052, Germany; 2 Department of Neurosurgery, University Hospital Regensburg, Franz-Josef-Strauß-Allee 11, Regensburg, 93052, Germany; 3 Department of Anesthesiology, Heart-Center Bad Krozingen, Südring 15, Bad Krozingen, 79189, Germany

## Abstract

**Background:**

Several techniques have been discussed as alternatives to the intermittent bolus thermodilution cardiac output (CO_PAC_) measurement by the pulmonary artery catheter (PAC). However, these techniques usually require a central venous line, an additional catheter, or a special calibration procedure. A new arterial pressure-based cardiac output (CO_AP_) device (FloTrac™, Vigileo™; Edwards Lifesciences, Irvine, CA, USA) only requires access to the radial or femoral artery using a standard arterial catheter and does not need an external calibration. We validated this technique in critically ill patients in the intensive care unit (ICU) using CO_PAC _as the method of reference.

**Methods:**

We studied 20 critically ill patients, aged 16 to 74 years (mean, 55.5 ± 18.8 years), who required both arterial and pulmonary artery pressure monitoring. CO_PAC _measurements were performed at least every 4 hours and calculated as the average of 3 measurements, while CO_AP _values were taken immediately at the end of bolus determinations. Accuracy of measurements was assessed by calculating the bias and limits of agreement using the method described by Bland and Altman.

**Results:**

A total of 164 coupled measurements were obtained. Absolute values of CO_PAC _ranged from 2.80 to 10.80 l/min (mean 5.93 ± 1.55 l/min). The bias and limits of agreement between CO_PAC _and CO_AP _for unequal numbers of replicates was 0.02 ± 2.92 l/min. The percentage error between CO_PAC _and CO_AP _was 49.3%. The bias between percentage changes in CO_PAC _(ΔCO_PAC_) and percentage changes in CO_AP _(ΔCO_AP_) for consecutive measurements was -0.70% ± 32.28%. CO_PAC _and CO_AP _showed a Pearson correlation coefficient of 0.58 (*p *< 0.01), while the correlation coefficient between ΔCO_PAC _and ΔCO_AP _was 0.46 (*p *< 0.01).

**Conclusion:**

Although the CO_AP _algorithm shows a minimal bias with CO_PAC _over a wide range of values in an inhomogeneous group of critically ill patients, the scattering of the data remains relative wide. Therefore, the used algorithm (V 1.03) failed to demonstrate an acceptable accuracy in comparison to the clinical standard of cardiac output determination.

## Background

Accurate evaluation of cardiac performance is an important goal in the treatment of critically ill patients. Usually a pulmonary artery catheter (PAC) is placed for assessment of cardiac output (CO), but as a result of the current discussion about the usefulness and risks of the PAC [1,2], several less invasive techniques have been discussed as alternatives to the intermittent bolus thermodilution cardiac output (CO_PAC_) measurement by the PAC [3], which still represents the clinical standard.

Particularly techniques using arterial waveform analysis for CO assessment (PiCCO*plus*^®^, Pulsion Medical Systems, Munich, Germany and LiDCO™*plus*, LiDCO Ltd, Cambridge, UK) have been tested as alternatives to the PAC, partly with excellent results [4-10]. However, these techniques usually require an additional catheter, a central venous line, or a special calibration procedure such as transpulmonary thermodilution (PiCCO*plus*^®^) or lithium dilution (LiDCO™*plus*). Furthermore, the inability of these devices to compensate for changes in individual aortic input impedance or central aortic compliance, which both may alter the calculated CO by the pulse contour technique in hemodynamic instability, can lead to erroneous assessment of CO [11,12].

A new arterial pressure-based cardiac output (CO_AP_) device (FloTrac™, Vigileo™; Edwards Lifesciences, Irvine, CA, USA) only requires access to the radial or femoral artery using a standard arterial catheter and does not need an external calibration. To facilitate CO_AP _assessment by arterial pulse waveform analysis without an external calibration mode, the system estimates individual arterial compliance according to Langewouters five component model [13] and continuously compensates for changes in vascular tone by detecting characteristic alterations in the arterial pressure waveform. But until now there has been only limited information about the value of this new device [14-16].

Therefore, this study was performed to evaluate the accuracy of FloTrac™-derived CO_AP _assessment in an inhomogeneous group of critically ill patients in a neurosurgical intensive care unit (ICU) using CO_PAC _as the method of reference.

## Methods

After obtaining approval of the Institutional Ethics Committee of the University of Regensburg Medical Centre (Regensburg, Germany) and with written informed consent from the patient or their relative, we studied 20 critically ill patients (10 male), aged 16 to 74 years (mean, 55.5 ± 18.8 years) in a neurosurgical ICU, who due to the severity of their illness required both, arterial and pulmonary artery pressure monitoring. Patients with intracardiac shunts or peripheral vascular disease were excluded from the study.

Analgesia based sedation was maintained with an infusion of fentanyl of 1.5–4.5 mg·kg^-1^·h^-1 ^and midazolam of 0.15–0.35 mg·kg^-1^·h^-1^, supplemented with an infusion of ketamine of 1.5–4.0 mg·kg^-1^·h^-1 ^in some patients. Pressure controlled ventilation (*BiLevel*^®^-mode, Bennett 840 ™ Ventilator System, Puritan Bennett, Pleasanton, CA, USA) with a positive end expiratory pressure of 5–15 mmHg and a tidal volume of 6–8 ml/kg to an end tidal pCO_2 _of 32–38 mmHg was maintained throughout the study.

All patients received a radial arterial line for continuous monitoring of arterial blood pressure (Siemens monitor SC 9000, Erlangen, Germany). A 7.5F pulmonary artery catheter (Baxter Healthcare Corporation, Irvine, CA, USA) was inserted via an 8.5 F introducer into the internal jugular or subclavian vein for intermittent thermodilution cardiac output (CO_PAC_) measurement (Siemens monitor SC 9000, Erlangen, Germany). CO_PAC _measurements were performed at least every 4 hours by injection of 10 ml iced saline solution via the CVP port of the PAC and subsequent detection by the thermistor embedded into the PAC. The average of 3 measurements, all measured within a 15% range randomly distributed over the respiratory cycle, was calculated according to the Stewart-Hamilton formula. If there was more than 15% variation between the values, five measurements were performed, the highest and lowest values from CO calculation were excluded, and the remaining three values averaged.

The FloTrac™sensor was attached to the existing arterial line and connected to the Vigileo™monitor for arterial pressure-based CO_AP _assessment. Following initiation of the Vigileo™monitor by entering patient's age, gender, height and weight, the system computes stroke volume (SV) from the patients arterial pressure signal and displays CO_AP _continuously.

The methodology of arterial pressure-based CO_AP _assessment by the FloTrac™system has been previously described by Manecke [17] and involves the calculation of SV regarding the proven relationship between pulse pressure (PP, the difference between systolic and diastolic blood pressure) and SV [18,19]. As demonstrated by Boulain, aortic PP is proportional to SV and is inversely related to aortic compliance for a given SV [18]. Based upon this physiological principle, the FloTrac™device assesses the arterial pulse waveform at a sampling rate of 100 Hz over a 20 second period, which generates approximately 2000 data points, and calculates the standard deviation (SD_AP_) of each measured beat to provide a robust assessment of key PP characteristics. Calculation of SD_AP _should be more precisely related to SV, in that multiple values are measured to determine the variability, rather than a simple single PP measurement [20]. SD_AP _of the arterial pressure waveform is computed on a beat-to-beat basis using the following equation:

SDAP=1N−1∑k=0N−1[AP(k)−APmean]2
 MathType@MTEF@5@5@+=feaafiart1ev1aaatCvAUfKttLearuWrP9MDH5MBPbIqV92AaeXatLxBI9gBaebbnrfifHhDYfgasaacPC6xNi=xI8qiVKYPFjYdHaVhbbf9v8qqaqFr0xc9vqFj0dXdbba91qpepeI8k8fiI+fsY=rqGqVepae9pg0db9vqaiVgFr0xfr=xfr=xc9adbaqaaeGacaGaaiaabeqaaeqabiWaaaGcbaGaem4uamLaemiraq0aaSbaaSqaaiabdgeabjabdcfaqbqabaGccqGH9aqpdaGcaaqaaKqbaoaalaaabaGaeGymaedabaGaemOta4KaeyOeI0IaeGymaedaaOWaaabCaeaacqGGBbWwcqWGbbqqcqWGqbaucqGGOaakcqWGRbWAcqGGPaqkcqGHsislcqWGbbqqcqWGqbaudaWgaaWcbaGaemyBa0MaemyzauMaemyyaeMaemOBa4gabeaakiabc2faDnaaCaaaleqabaGaeGOmaidaaaqaaiabdUgaRjabg2da9iabicdaWaqaaiabd6eaojabgkHiTiabigdaXaqdcqGHris5aaWcbeaaaaa@50B3@

where AP(k) is the k^th ^pulse pressure sample in the current beat, N is the total number of samples, and AP_mean _is the mean of arterial pressure [21]. To continuously compensate for changes in vascular compliance and peripheral resistance, a scale factor χ is calculated on the basis of biometric data [13] and the analysis of characteristic alterations of the individual arterial pressure waveform, such as skewness or kurtosis, reflecting changes in vascular tone. In the used version of software (V 1.03), χ was recalculated every 10 minutes. Thus, CO_AP _is computed as:

*CO*_AP _= *HR *• *SD*_AP _• *χ*

where HR is the heart rate and χ a scale factor proportional to vascular compliance and peripheral resistance.

FloTrac™-derived CO_AP _values were taken immediately at the end of bolus determinations, representing an average over the last minutes. Measurements were completed when the PAC or the arterial catheter was removed, or the patient was weaned from mechanical ventilation.

### Statistical Analysis

The bias between CO_PAC _and CO_AP _for all values and for percentage changes (Δ = trend analysis) between consecutive CO determinations was calculated as the mean difference between measurements and expresses the agreement between methods. To avoid a systematical calculation error in the case of repeated measurements per subject, the bias between methods was calculated according to a modified statistical approach by Bland and Altman for unequal numbers of replicates [22]. The upper and lower limits of agreement (LOA), defining the range in which 95% of the differences between methods are expected to lie, were calculated as bias ± 1.96 SD. The bias and the LOA are reported as 95% confidence interval. The percentage error, defined as the standard deviation of the bias (± 1.96 SD) divided by the mean CO, was calculated according to Critchley and Critchley [23]. The acceptable limit of variability between techniques was determined as a percentage error below ± 30%. Linear regression analysis was performed between the absolute values of CO_PAC _and CO_AP _and between percentage changes in CO_PAC _and CO_AP_. A p < 0.05 was regarded as significant.

## Results

Demographic data, patient characteristics and the number of measurement repetitions per patient are presented in Table 1.

**Table 1:** Demographic data and patient characteristics

Patient	Diagnosis	Age (years)	Sex	Apache II Score	BMI (kg/m^2^)	Measurements (n)
1	ICB, Acute heart failure	70	female	37	34.3	20
2	Cerebral infarct, Angina pectoris	73	female	36	27.3	6
3	Craniocerebral injury	21	male	14	27.7	5
4	SAH	46	female	12	19.6	7
5	Craniocerebral injury, Thorax trauma	18	male	28	19.0	8
6	SAH, Cerebral infarct	49	female	26	25.8	8
7	Sepsis	62	male	37	31.4	6
8	SAH	66	male	21	28.4	15
9	PAH	71	female	17	28.6	6
10	ICB, Hypertension	49	female	21	24.5	14
11	Craniocerebral injury, Cardiopulmonary resuscitation	16	female	31	23.9	7
12	Coronary heart disease, CABG	71	male	29	31.1	5
13	Mitral regurgitation, MVR	74	male	37	33.0	4
14	SAH, ICB	43	female	23	22.8	13
15	Coronary heart disease, Mitral regurgitation, CABG, MVR	64	male	12	22.4	3
16	Coronary heart disease, CABG	69	male	20	28.3	8
17	Cerebral infarct	73	female	32	35.9	7
18	SAH, Pulmonary edema	53	male	27	26.4	8
19	Intracranial haematoma	70	male	20	33.1	11
20	Sepsis, Cardiopulmonary resuscitation	52	female	34	24.2	3

all		55.5 ± 18.9	10 male	25.7 ± 8.4	27.4 ± 4.7	164

Data are presented as mean ± SD or as frequency distributions (n) and simple percentages (%).

Appreviations: BMI = body mass index; CABG = coronary artery bypass grafting; ICB = intracerebral bleeding; MVR = mitral valve replacement; PAH = pulmonary arterial hypertension; SAH = subarachnoidal haemorrhage.

**Figure 1:**
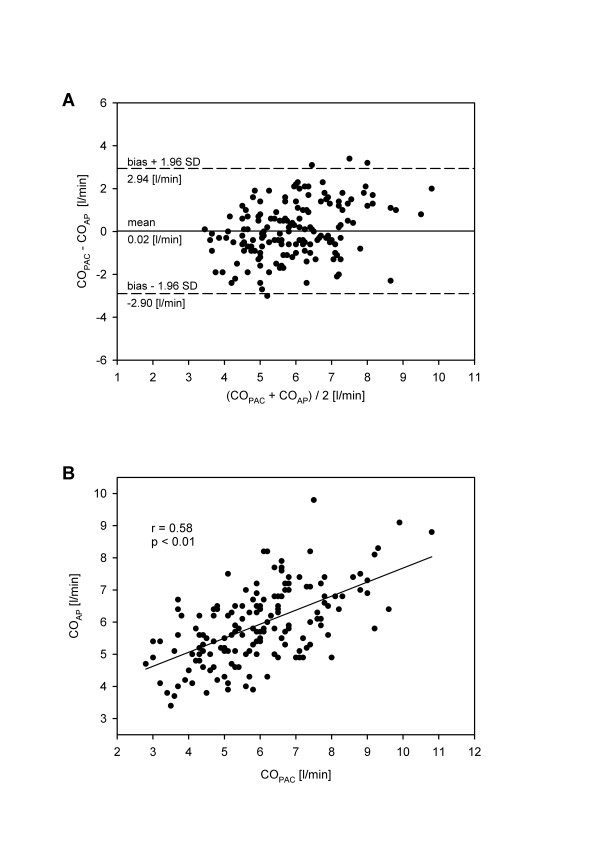
Bland-Altman plot **(A) **and regression analysis **(B) **for comparison between the intermittent thermodilution-derived cardiac output (CO_PAC_) by the pulmonary artery catheter (PAC) and the arterial pressure-based cardiac output (CO_AP_) by the FloTrac™system for unequal numbers of replicates. The solid line represents the mean difference (bias) and the dashed lines represent the limits of agreement (bias ± 1.96 SD).

A total of 164 coupled measurements were obtained. Absolute values of CO_PAC _ranged from 2.80 to 10.80 l/min (mean 5.93 ± 1.55 l/min), while absolute values of CO_AP _ranged from 3.40 to 9.80 l/min (mean 5.91 ± 1.15 l/min). The modified Bland-Altman analysis for an unequal number of replicates between CO_PAC _and CO_AP _showed a mean bias and LOA of 0.02 ± 2.92 l/min (Figure 1A). The percentage error between CO_PAC _and CO_AP _was 49.3%. CO_PAC _and CO_AP _showed a correlation coefficient of 0.58 (*p *< 0.01) as displayed in Figure 1B.

**Figure 2:**
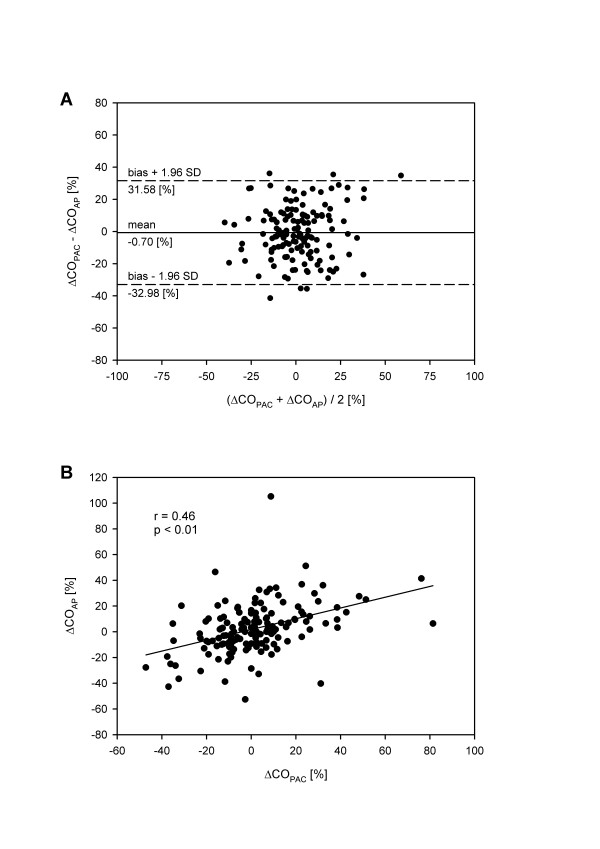
Bland-Altman plot **(A) **and regression analysis **(B) **for comparison between the percentage changes in intermittent thermodilution-derived cardiac output (ΔCO_PAC_) by the pulmonary artery catheter (PAC) and arterial pressure-based cardiac output (ΔCO_AP_) by the FloTrac™system for consecutive measurements. The solid line represents the mean difference (bias) and the dashed lines represent the limits of agreement (bias ± 1.96 SD).

A total of 144 coupled measurements were obtained for a trend analysis of percentage changes in CO. The bias between percentage changes in CO_PAC _(ΔCO_PAC_) and percentage changes in CO_AP _(ΔCO_AP_) for consecutive measurements was -0.70% with LOA of ± 32.28% (Figure 2A). ΔCO_PAC _and ΔCO_AP _revealed a correlation coefficient of 0.46 (*p *< 0.01) as shown in Figure 2B.

The results of the analysis of agreement, assessed by bias, and the distribution of the observed differences, indicated by bias ± 1.96 SD as upper and lower LOA including 95% confidence intervals are presented in Table 2.

**Table 2:** Mean difference (bias) between intermittent thermodilution cardiac output (CO_PAC_) and arterial pressure-based cardiac output (CO_AP_) for all values (A) and for percentage changes between consecutive measurements of CO_PAC_ (ΔCO_PAC_) and CO_AP_ (ΔCO_AP_) (B) according to the modified approach by Bland and Altman for unequal numbers of replicates with upper and lower limits of agreement (bias ± 1.96 SD), together with 95% confidence intervals (in parentheses)

**A**	CO_PAC _[l/min]	CO_AP _[l/min]	Bias (95% CI) [l/min]	Upper limit of agreement (95%CI) [l/min]	Lower limit of agreement (95%CI) [l/min]
	
	5.93 ± 1.55	5.91 ± 1.15	0.02 (-0.40 to 0.44)	2.94 (1.81 to 4.07)	-2.90 (-1.77 to 4.03)
**B**	ΔCO_PAC _[%]	ΔCO_AP _[%]	Bias (95% CI) [%]	Upper limit of agreement (95%CI) [%]	Lower limit of agreement (95%CI) [%]
	
	0.91 ± 18.84	1.61 ± 17.08	-0.70 (-3.45 to 2.05)	31.58 (26.85 to 36.30)	-32.98 (-37.70 to -28.26)

## Discussion

This should be the first investigation evaluating a new arterial pressure-based CO-device in an inhomogeneous group of critically ill patients on a neurosurgical ICU in comparison to the clinical standard of CO determination. The fact that all patients received pulmonary artery pressure monitoring due to the severity of their illness differentiates our trial from previous studies, which all evaluated FloTrac-system in elective cardiac surgical patients, representing a relatively homogeneous, hemodynamically stable and artificial group of patients in which pulmonary artery pressure monitoring is not necessarily performed.

The results of the present study demonstrate a minimal bias between the absolute values of CO_PAC _and CO_AP _over a large range of values in an inhomogeneous group of critically ill patients. However, a relative wide scattering of the data could be observed, possibly due to the calculation mode with which the algorithm compensates for changes in vascular tone.

Several studies have shown that less invasive devices for CO assessment based on arterial waveform analysis, such as PiCCO*plus*^® ^or LiDCO™*plus*, are valuable alternatives to the intermittent thermodilution technique by the PAC [4-10]. However, all these techniques require an additional catheter, a central venous line, or an external calibration procedure. Furthermore, the inability of these techniques to continuously compensate for changes in vascular tone during hemodynamic instability [12] with the consequential need of frequent recalibrations invalidates strictly speaking the claim of a continuously measuring device. Therefore, finding a minimal invasive device for accurate and continuous CO assessment, which is simple to handle and independent of an additional catheter and an external calibration mode, is still of constant clinical interest.

The recently introduced FloTrac™system should be an interesting alternative for CO assessment and may have potential advantages due to its relative non-invasiveness and simplicity, but until now there has been only limited information about the value of this new device [14-16]. Opdam and colleagues recently studied six patients after elective cardiac surgery and stated that the cardiac index (CI) values obtained with the FloTrac™system were imprecise compared with the PAC because of an inconsistent bias (0.21 l·min^-1^·m^2^) and a wide scattering of data (± 1.02 l·min^-1^·m^2^) [15]. However, major limitations of that study were the small number of patients included (6 patients), the extremely varying replicates of CO determinations obtained for each patient (8 *vs*. 158) and an inadequate statistical method. Not using the modified statistical approach by Bland and Altman for unequal numbers of replicates in comparison studies may falsify any result in an unpredictable manner. Variability of measurements made on different subjects is usually much greater than the variability between measurements on the same subject, and this phenomenon should be taken into account. In the worst case the findings may merely reflect the results of the patient with the majority of measuring repetitions.

Sander and co-workers compared 30 CABG patients at four different time points and demonstrated that CO measurements by the FloTrac™system showed a high bias (0.6 l/min, with a percentage error of 54%) and a wide range of LOA (± 2.8 l/min) in comparison with the CO_PAC _measurement [16]. The authors suggest that CO assessment with pulse contour analysis techniques in a setting after cardiac surgery basically might not be the ideal method. Similar results were recently reported by Mayer et al, who found a bias and LOA of 0.46 ± 1.15 l·min^-1^·m^2 ^with a percentage error of 46% for CI comparisons between methods in 40 cardiac surgical patients [14].

Our results are in accordance with the findings of the very limited number of publications regarding the accuracy of CO_AP _assessment and also revealed a relative wide scattering of data as shown in Figure 1A. All these investigations were performed with the same version of FloTrac™system software (V 1.03) in which the algorithm compensates for changes in vascular resistance using a 10 minute moving average. Obviously, this method may have difficulty responding to rapid changes in vascular tone during hemodynamic instability. Improvement regarding the response time to changes in vascular tone has been accomplished in a subsequent version of software (V 1.07). The 10 minute moving window for assessing vascular tone was set to a 1 minute moving window.

The estimation of CO_PAC _has become the clinical standard, despite the fact that the method has limited accuracy and may be affected by many factors such as ventilation, volume and temperature of injectate, and technique of indicator injection [24,25], which may have contributed to the variations found between methods in the present study. Differences of at least 15% in CO_PAC _must be achieved under clinical conditions for clinical relevance when using the triplicate method [24,26]. Critchley and Critchley demonstrated that errors of both test and reference method should be combined when assessing comparative CO, which results in a percentage error of ± 30% to be clinically acceptable [23]. The percentage error of ± 49.3% between CO_PAC _and CO_AP _found in the present study exceeded the recommended limit to accept a new technique that has been compared to the reference method.

Accuracy of CO assessment is an important issue and should certainly be comparable between methods, but the more relevant question for a clinician, operating with the FloTrac™system in an ICU setting, concerns the value of CO_AP _assessment to reasonably detect the direction of changes in CO under different hemodynamic conditions. Trend analysis of percentage changes in CO between consecutive measurements (Figure 2A) demonstrates a minimal bias between ΔCO_PAC _and ΔCO_AP _and LOA slightly above the value suggested by Critchley and Critchley [23]. While the absolute values of CO did not show an exact agreement between methods, the direction of changes in CO_PAC _assessment was obviously identified more precisely by the FloTrac™system. However, to demonstrate that the CO_AP _algorithm correctly identifies changes in vascular tone, interventional studies are required in which the FloTrac™system has to be compared to a continuous measuring gold standard.

The range of cardiac outputs in the present investigation is large, which should add power to our findings as all often comparable studies were performed on hypodynamic groups of patients. However, analysing the Bland-Altman plot (Figure 1A), a slight trend for the FloTrac™system to underestimate high CO values and to overestimate low CO values could be observed. This should be taken into account for further improvements of the algorithm.

Even though the FloTrac™system was designed as a screening tool in a segment of patients, who might have been under-monitored before, any new CO device has to be compared to the clinical gold standard of CO determination by the PAC in varying clinical situations before it could be recommended for a broader application. Once attached to a pre-existing arterial line, the device provides additional information within 20 seconds without any external calibration procedure, which may be valuable in the care of an endangered patient. The advantages of the device compared to other monitoring systems using the arterial pressure for CO assessment are rationalized by its simplicity and its lack of necessity for a time-consuming placement of an additional catheter.

## Limitations

The results of the present study are limited by the small and varying number of CO estimations obtained for each patient. However, even in this small group of patients, the findings clearly demonstrate the limitations of the new device and the need for improvements regarding the response time to rapid changes in vascular tone. Furthermore, as changes in vascular tone have not been induced in this investigation, additional interventional studies are required to compare the latest version of FloTrac™software to a continuous measuring gold standard regarding its ability to correctly identify changes in arterial compliance.

## Conclusion

FloTrac™system has the potential to be a promising alternative for cardiac output measurement, but the used algorithm (V 1.03) failed to demonstrate an acceptable accuracy in comparison to the clinical standard of CO determination. Further studies are required to evaluate the accuracy of this new device in various experimental and clinical settings using the latest version of software.

## List of abbreviations

AP = arterial pressure; BMI = body mass index; CABG = coronary artery bypass grafting; CO = cardiac output; CO_AP _= arterial pressure-based cardiac output; CO_PAC _= intermittent bolus thermodilution cardiac output; CVP = central venous pressure; HR = heart rate; ICB = intracerebral bleeding; ICU = intensive care unit; LOA = limits of agreement; MVR = mitral valve replacement; PAC = pulmonary artery catheter; PAH = pulmonary arterial hypertension; PP = puls pressure; s = seconds; SAH = subarachnoid haemorrhage; SD = standard deviation; SD_AP _= standard deviation of the arterial pressure; SV = stroke volume; χ = scale factor proportional to vascular compliance and peripheral resistance.

## Competing interests

CW received a travel grant from Edwards Lifesciences (Irvine, USA) to present this data at an international meeting. The authors declare that there are no further competing interests.

## Authors' contributions

CP designed the study and performed the statistical analysis.

SB collected the clinical data and participated in the design of the study.

CK extensively revised the manuscript.

SS collected the clinical data and participated in the analysis of the data.

BT collected and processed the clinical data.

MA collected the clinical data and participated in the analysis of the data.

JW collected the clinical data and participated in the analysis of the data.

CW designed the study, processed the data and wrote the manuscript.

All author(s) read and approved the final manuscript.

## Pre-publication history

The pre-publication history for this paper can be accessed here:


